# Triggers for an episode of sudden onset low back pain: study protocol

**DOI:** 10.1186/1471-2474-13-7

**Published:** 2012-01-24

**Authors:** Daniel Steffens, Manuela L Ferreira, Christopher G Maher, Jane Latimer, Bart W Koes, Fiona M Blyth, Paulo H Ferreira

**Affiliations:** 1 Musculoskeletal division, The George Institute for Global Health, Sydney Medical School, The University of Sydney, PO Box M201, Missenden Road, Sydney, New South Wales 2050, Australia; 2 Department of General Practice, Erasmus MC, PO Box 2040, 3000 CA Rotterdam, The Netherlands; 3 Centre for Education and Research on Ageing, The University of Sydney, C 22 - Concord Hospital, Sydney, New South Wales 2006, Australia; 4 Discipline of Physiotherapy, Faculty of Health Sciences, The University of Sydney, PO Box 170, Lidcombe 1825, Syndey, New South Wales, Australia

## Abstract

**Background:**

Most research on risk factors for low back pain has focused on long term exposures rather than factors immediately preceding the onset of low back pain. The aim of this study is to quantify the transient increase in risk of a sudden episode of low back pain associated with acute exposure to a range of common physical and psychological factors.

**Methods/design:**

This study uses a case-crossover design. One thousand adults with a sudden onset of low back pain presenting to primary care clinicians will be recruited. Basic demographic and clinical information including exposure to putative triggers will be collected using a questionnaire. These triggers include exposure to hazardous manual tasks, physical activity, a slip/trip or fall, consumption of alcohol, sexual activity, being distracted, and being fatigued or tired. Exposures in the case window (0-2 hours from the time when participants first notice their back pain) will be compared to exposures in two control time-windows (one 24-26 hours and another 48-50 hours before the case window).

**Discussion:**

The completion of this study will provide the first-research based estimates of the increase in risk of a sudden episode of acute low back pain associated with transient exposure to a range of common factors thought to trigger low back pain.

## Background

Nearly 4 million people in Australia suffer from back pain at any one time [1], with total treatment costs exceeding $1 billion a year [2]. In the US, the figure is an astonishing US$32 billion a year [3]. Back complaints are the seventh most common condition in patients consulting general practitioners in Australia, and the most common musculoskeletal condition [4]. It is also the most common health problem for which an imaging test is ordered by a general practitioner [4].

A potential solution to managing the problem of low back pain is the identification and control of modifiable risk factors. This approach is appealing and seemingly logical and there are notable examples where such an approach has provided major improvements in public health. For back pain this approach has not yet been fruitful: Cochrane reviews of workplace interventions [5], insoles [6] and lumbar supports [7] have failed to support these traditional back pain prevention approaches. Prevention strategies have to date been largely based on controlling long-term exposure to risk factors, for example, modifying seats to control vibration in truck drivers. However it is likely that the full potential of prevention will not be reached unless we also consider commonly occurring, modifiable risk factors that happen just before the onset of back pain. In this regard we see our proposed research as complementary to, rather than in conflict with, research evaluating long term risk factors.

The existence of short term risk factors or 'triggers' is consistent with the time course of back pain. It is well established that most people will experience low back pain in their lifetime [8], that pain is typically recurrent [9] and that episodes are usually of sudden onset [10]. For example research conducted by our group demonstrated that in an inception cohort of 969 subjects, 82% reported that their onset of low back pain was sudden [10]. This pattern of low back pain suggests that rather than solely looking at long term exposure to risk factors it would be instructive to also look closely at events occurring immediately prior to the episode to identify modifiable triggers to the episode. This information is routinely sought by health practitioners when a patient with low back pain seeks care. The treating clinician commonly asks the patient what activity they were performing just prior to the onset of pain, in essence, "was the episode triggered by something unusual that happened just before?" The scientific method best suited to answer this question is the case-crossover design [11].

We will use the case-crossover design to provide the first accurate estimates of the transient increase in risk of low back pain associated with transient exposure to various triggers. It is possible that we will identify several factors that are not modifiable but this information will be extremely important to our understanding and explanation of the causes of low back pain.

### Study Aims

1) To quantify the transient increase in risk of an episode of sudden onset, acute, low back pain associated with exposure to a range of common physical and psychological factors listed in Table 1.

**Table 1:** Factors that may trigger an episode of low back pain to be evaluated in the study

*Physical Factors*	*Hazardous manual tasks:*
	- tasks involving heavy loads;
	- tasks involving awkward postures;
	- tasks involving objects that could not be positioned close to the body;
	- tasks involving live people or animals;
	- tasks involving loads that are unstable, unbalanced or difficult to grasp or hold;
	
	*Vigorous physical activity*
	
	*Moderate physical activity*
	
	*A slip/trip or fall*
	
	*Consumption of alcohol*
	
	*Sexual activity*
***Psychological***	*Being distracted*
	
***Factors***	*Being fatigued or tired*

2) To determine if habitual physical activity moderates the transient increase in risk of an episode of sudden onset, acute, low back pain associated with exposure to the physical and psychological factors listed above.

## Methods/Design

The study will use the case-crossover design. The case-crossover design enables quantification of the risk associated with transient exposures [12]. It is more efficient than cohort designs because it samples only cases, and may be less exposed to selection bias than case-control designs because cases provide their own control data. Cases will be identified from patients presenting to primary care seeking treatment for an episode of sudden onset, acute, low back pain. In the case crossover design the time of the onset of low back pain is identified and then data are obtained on exposure to a series of possible risk factors in the two hour period prior to the onset of low back pain (case window). Additional data are obtained on exposures to the same set of possible risk factors in an earlier period (24-26 and 48-50 hours prior to the case window) that did not precede an episode of low back pain (these are referred to as the control windows).

The study has been approved by the Human Research Ethics Committee at the University of Sydney (protocol number 05-2011/13742) and has received funding from Australia's National Health and Medical Research Council (application ID APP1003608).

### Study Participants

One thousand consecutive patients (study participants) presenting to primary care clinicians (general medical practitioners, physiotherapists, chiropractors and pharmacists) for treatment of an episode of sudden onset, acute, low back pain will be recruited in Sydney, Australia. Primary care clinicians will be trained individually or in small groups on the study methods and procedures. Study participants must be 18 years of age (or older) to participate.

To be eligible to enter the study participants must meet the criteria below:

▪ Comprehends spoken English;

▪ Primary complaint of pain in the area between the 12^th ^rib and buttock crease, with or without leg pain;

▪ Pain at least moderate intensity during the first 24 hours of the episode (assessed using a modified version of item 7 of the SF36);

▪ Presentation for treatment within 7 days from the time of pain onset;

▪ Not have known or suspected serious spinal pathology (eg metastatic, inflammatory or infective diseases of spine, cauda equina syndrome, spinal fracture);

An episode of acute low back pain will be defined as an episode preceded by a period of at least one month without low back pain where the participant was not consulting a health care practitioner or continuing with medication for their low back pain (in accordance with the De Vet et al. [13] definition of an 'episode' of acute low back pain). A sudden onset episode of low back pain will be defined as pain of at least moderate intensity that developed over the first 24 hours (assessed using a modified version of item 7 of the SF36).

To describe further the cohort of study clinicians, we will collect descriptive data, including the clinician's age, contact details, current position and past clinical experience. Secondly, we are collecting information regarding what clinicians in general consider as possible triggers for a new episode of back pain. Based on their clinical experience, they are asked to list the five most likely triggers for a sudden onset episode of low back pain. They will consider both (i) short term and (ii) long term exposures (see additional file 1). These data will be used to inform the categorisation of putative risk factors in the analyses of our participant data, and to assess whether opinions of the study participants regarding possible triggers for their low back pain are analogous with their clinicians' perceptions.

### Participant recruitment

Patients seeking care for acute low back pain that fulfil the inclusion criteria and agreeing to participate will be referred to the study and their details (screening form and consent form) will be sent by fax to the study office. A study researcher will receive the fax and contact the participant as soon as possible to perform the study interview. Patients not able to answer the study questionnaire in seven days from the time their clinician referred them to the study will be excluded.

Prior to the study interview, the researchers will double-check the eligibility criteria and explain the nature of the study to the participant. Participants are able to withdrawn from the study at any time.

### Study interview

The interview is divided into two parts. In the first part we will collect basic demographic and clinical data and in the second part, we will collect information on putative triggers (see additional file 2). We will record the date and time when the patient first noticed their back pain. Where possible, using a diary, calendar and/or smartphone, we will then ask them to recall what they were doing in the three days leading up to the onset of their back pain and also on the day of their back pain.

Following this we will ask about exposure to the previously mentioned putative triggers. Where subjects respond affirmatively we collect detailed information on the trigger, time and duration in free text. We will also ask the study participant to consider what they think may have triggered their LBP and similarly record detailed information on the nominated trigger, time and duration in free text.

When asking the study participant about exposure to specific triggers we have developed a script to lead the interview (see additional file 2).

### Blinding

Clinicians and study participants will be blinded to the case and control periods. The study questionnaire is designed to investigate exposure to triggers over a longer time period than will be used in the analysis so that participants in the trial remain blind to the duration of the case and control windows. For example participants will be asked about their exposures for three days preceding their back pain and also on the day of their back pain. A random sample of telephone calls will be audited and the congruency of the log and telephone call checked by the investigators. Data entry into the database will be conducted by a separate person who will be blinded to all putative risk factors. Blinding may be less important in case-crossover designs than case-control studies because in the case-crossover design participants report exposure to triggers in *both *the case and control windows. Recall bias can only occur if there is *differential *mis-reporting in the case and control windows. In our opinion this is unlikely.

### Statistical analysis

The analyses follows standard methods for stratified analyses [12] with the individual subject the stratifying variable in a case-crossover design. The estimates of relative risk are based on the ratio of the observed frequency of exposure to each of the transient triggers during the case period, to the expected frequency of exposure during the two control periods. This is known as a matched-pair interval approach where contrasts are made between a pair of case control periods contributed by the same subject. In our proposed study there will be two matched-pair intervals.

To analyse the matched-pair interval data we will use standard methods for case-control data (Mantel-Haenszel estimator). Instead of having concordant and discordant pairs of subjects, the pairs will consist of two intervals for each subject, a case period (2 hours prior to the event) and a control period (24-26 hours prior to the event). A subject's pair of intervals will either be concordant or discordant with respect to each of the triggers nominated on the item list. Ninety-five percent confidence intervals will be computed by exact methods based on the binomial distribution. Comparison with the first control period will form the primary analyses. Secondary analyses will be performed as described above but using the second control period (48-50 hours prior to the event) as the control data for the matched-pair interval.

### Sample size

The study was designed to be adequately powered for the primary analysis, which involves estimation of the risk associated with transient exposure to the different types of triggers. We calculated the sample size necessary for a paired case-control study using the procedures described by Dupont [14]. This showed that in a conventional paired case-control design with alpha set at 0.05 we would need a sample of 1,000 cases to provide an 80% probability of detecting an odds ratio of 1.5 or greater across the plausible range of exposure prevalence's in control windows (0.2 to 0.8) and plausible range of correlations between exposure in case and control windows (0.0 to 0.5).

## Discussion

This case cross-over study will provide the first-research based estimates of the transient increase in risk of a sudden onset, acute, episode of low back pain associated with transient exposures to a range of physical and psychological factors. We anticipate that we will identify several modifiable factors that are triggers for an episode of low back pain. This information will be invaluable in designing future prevention strategies, and enabling clinicians to give evidence based advice to patients keen to avoid future episodes of low back pain.

Recall bias is a major limitation of retrospective studies. Participants may under or overestimate the usual frequency of exposures to the time of the injury (case window). They may also under or overestimate exposure in the case control windows because of memory lapse or difficulty in estimating exposure. In this study, participants must have presented for care within seven days of the onset of the injury to facilitate recall of activities. In addition, our questionnaire asks participants to use prompts such as referring to their agenda, calendar and/or smartphones to stimulate their memory of the activities they performed in the days prior to the onset of their low back pain.

The completion of this trial is expected by early 2014.

## Competing interests

The authors declare that they have no competing interests. Prof Maher and A/Prof Latimer's fellowships are funded by the Australian Research Council.

## Authors' contributions

CGM, JL, MLF, BWK, FMB and PHF are the principal investigators - together they conceived and designed the trial and procured funding. DS and MLF drafted the first version of the manuscript. All authors contributed to the writing of the manuscript. All authors read and approved the final version of the manuscript.

## Pre-publication history

The pre-publication history for this paper can be accessed here:

http://www.biomedcentral.com/1471-2474/13/7/prepub

## Supplementary Material

Additional file 1 **Clinicians' questionnaire**. Questionnaire to be applied to describe further the cohort of study clinicians. Click here for file

Additional file 2 **Study Participants' Questionnaire**. Questionnaire to be applied to the study participants. Click here for file

## References

[B1] Australian Institute of Health and Welfare Arthritis and musculoskeletal conditions in Australia 2005 Canberra: Australian Institute of Health and Welfare

[B2] Walker B Muller R Grant W Low back pain in Australian adults: the economic burden Asia Pac J Public Health 2003 15 2 79 87 10.1177/101053950301500202 15038680

[B3] Agency for Healthcare Research and Quality Total Expenses and Percent Distribution for Selected Conditions by Type of Service: United States 2005 Medical Expenditure Panel Survey Household Component Data

[B4] Britt H Miller G Knox S Charles J Pan Y Henderson J Baryram C Valenti L Ng A O'Halloran J General practice activity in Australia 2004-2005 2005 Canberra: Australian Institute of Health and Welfare

[B5] Van Oostrom S Driessen M de Vet H Franche R Schonstein E Loisel P Van Mechelen W Anema J Workplace interventions for preventing work disability Cochrane Database of Systematic Reviews 2009 10.1002/14651858.CD006955.pub2 19370664

[B6] Sahar T Cohen MJ Ne'eman V Kandel L Odebiyi D Lev I Brezis M Lahad A Insoles for prevention and treatment of back pain Cochrane Database of Systematic Reviews 2007 4 CD005275 10.1002/14651858.CD005275.pub2 PMC13187884 17943845

[B7] Van Duijvenbode I Jellema P Van Poppel MN Van Tulder MW Lumbar supports for prevention and treatment of low back pain Cochrane Database of Systematic Reviews 2008 2 10.1002/14651858.CD001823.pub2 17636685

[B8] Waddell G The Back Pain Revolution 2004 2 Edinburgh: Churchill Livingstone

[B9] Von Korff M Saunders K The course of back pain in primary care Spine 1996 21 24 2833 2839 10.1097/00007632-199612150-00004 9112707

[B10] Henschke N Maher CG Refshauge KM A systematic review identifies five red flags to screen for vertebral fracture in patients with low back pain J Clin Epidemiol 2008 61 2 110 118 10.1016/j.jclinepi.2007.04.013 18177783

[B11] Maclure M Mittleman M Should we use a case-crossover design? Annu Rev Public Health 2000 21 193 221 10.1146/annurev.publhealth.21.1.193 10884952

[B12] Maclure M The case-crossover design: a method for studying transient effects on the risk of acute events Am J Epidemiol 1991 133 144 153 1985444 10.1093/oxfordjournals.aje.a115853

[B13] de vet H Heymans M Dunn K Pope D van der Beek A Macfarlane G Bouter L Croft P Episodes of low back pain. A proposal for uniform definitions to be used in research Spine 2002 27 21 2409 2416 10.1097/00007632-200211010-00016 12438991

[B14] Dupont W Power calculations for matched case-control studies Biometrics 1988 44 1157 1168 10.2307/2531743 3233252

