# Supplementary material for: Triggers for an episode of sudden onset low back pain: study protocol
Source: BMC Musculoskelet Disord. 2012 Jan 24;13:7. doi: 10.1186/1471-2474-13-7 (PMC3292970; doi:10.1186/1471-2474-13-7)
Supplement: Additional file 1 — Clinicians' questionnaire. Questionnaire to be applied to describe further the cohort of study clinicians. [file 1471-2474-13-7-S1.DOCX]

**Name**: ___________________________________________________________________

**Additional file 1 – Clinicians’ Questionnaire**


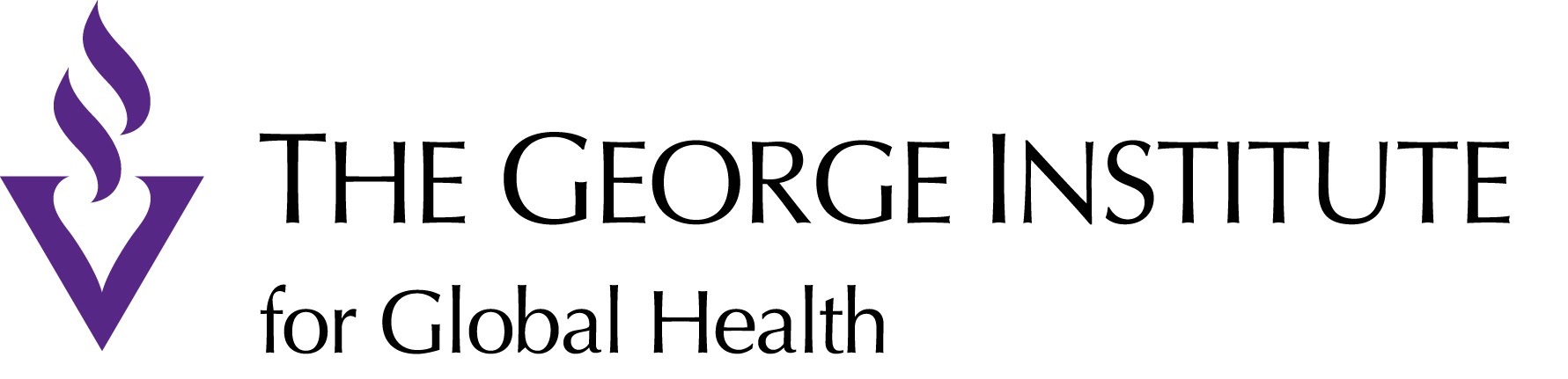


**Gender**: □ Female □ Male **Date of Birth**: _____________________________

**Address**: ________________________________________________________________

**Phone**:_____________________________ **FAX**: ________________________________

**Email**:_____________________________

**Profession (tick one)**

□ physiotherapist □ medical practitioner □ pharmacist

**Current Position**:__________________________________________________________________

**Clinical experience**

Years as practicing clinician _______________________________

Years managing low back pain ______________________________

**1. Based on your clinical experience, list what you consider to be the five most likely factors involving short term exposure that are triggers for a sudden episode of acute low back pain?** (E.g. in my clinical experience, running 15km on the road with poor shoes can trigger an episode of shin splints).

**1)**________________________________________________________________________**2)**________________________________________________________________________ **3)**________________________________________________________________________**4)**________________________________________________________________________ **5)**________________________________________________________________________

**2. Based on your clinical experience, list what you consider to be the five most likely factors involving long term exposure that increase the risk of a sudden episode of acute low back pain** (e.g. in my clinical experience working with a ‘poke neck’ posture increases the risk of neck pain and headaches).

**1)**________________________________________________________________________**2)**________________________________________________________________________ **3)**________________________________________________________________________**4)**________________________________________________________________________ **5)**________________________________________________________________________
