# Supplementary material for: Triggers for an episode of sudden onset low back pain: study protocol
Source: BMC Musculoskelet Disord. 2012 Jan 24;13:7. doi: 10.1186/1471-2474-13-7 (PMC3292970; doi:10.1186/1471-2474-13-7)
Supplement: Additional file 2 — Study Participants' Questionnaire. Questionnaire to be applied to the study participants. [file 1471-2474-13-7-S2.DOCX]

**Triggers Specific Questions**

**Additional file 2 – Study Participants’ Questionnaire**

Tick the boxes where the participant reports pain on the mannequin below:


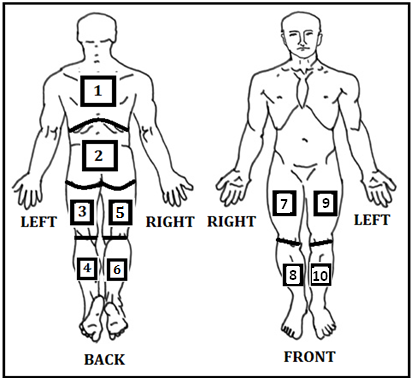


**Height** (cm): ______________________ **Weight** (Kg): _____________________

**How much back pain have you had during the first 24 hours of this episode**? [1]

| None | Very mild | Mild | Moderate | Severe | Very Severe |
| --- | --- | --- | --- | --- | --- |
| 🌕 | 🌕 | 🌕 | 🌕 | 🌕 | 🌕 |

**During the first 24 hours of this episode how much did back pain interfere with your normal work** (including both work outside the home and housework)? [1]

| Not at all | A little bit | Moderately | Quite a bit | Extremely |
| --- | --- | --- | --- | --- |
| 🌕 | 🌕 | 🌕 | 🌕 | 🌕 |

**How tense or anxious have you felt in the past week? Circle one.** [2]

| 0  Calm and relaxed | 1 | 2 | 3 | 4 | 5 | 6 | 7 | 8 | 9  As tense/anxious as I’ve ever felt | 10 |
| --- | --- | --- | --- | --- | --- | --- | --- | --- | --- | --- |

**Physical Activity** [3]

**1.** In **the last week**, how many times have you walked continuously, for at least 10 minutes, for recreation, exercise or to get to or from places? ______times;

And in **the week before** your low back pain started? ______times.

What do you estimate was the total time that you spent walking in this way in the last week? ______hours ______minutes;

And in the week before your low back pain started? ______ hours ______minutes.

**2.** In **the last week**, how many times did you do any vigorous physical activity which made you breathe harder or puff and pant? (e.g. jogging, cycling, aerobics, competitive tennis)______times;

And **in the week before** your low back pain started? ______times.

What do you estimate was the total time that you spent doing this vigorous physical activity in the last week? ______hours ______minutes;

And in the week before your low back pain started? ______ hours ______minutes.

**3.** In **the last week**, how many times did you do any moderate physical activities that you have not already mentioned? (e.g. gentle swimming, social tennis, golf)______times;

And **in the week before** your low back pain started? ______times.

What do you estimate was the total time that you spent doing these activities in the last week? ______hours ______minutes;

And in the week before your back pain started? ______ hours ______minutes.

**4.** Was your level of physical activity **last week** typical for you? □ Yes □ No

**Triggers Exposure**

**Please write the date and time you first noticed your low back pain on the table below:**

| **Day** | **Fri** | **Sat** | **Sun** | **Mon** | **Tues** | **Wed** | **Thurs** | **Fri** | **Sat** | **Sun** |
| --- | --- | --- | --- | --- | --- | --- | --- | --- | --- | --- |
| **Date** |  |  |  |  |  |  |  |  |  |  |
| **Time** |  |  |  |  |  |  |  |  |  |  |

**I am going to ask you to recall what you were doing in the three days leading up to your back pain and also on the day of your back pain. To help your memory I would like you to sit down with your diary and smartphone. To help make sure we have the right days I want you to tell me the day, weather and a key thing you did on each day.**

Example: Tuesday: cold and wet; visited parents

Day of back pain: _______________________________________________________

Day before: ____________________________________________________________

2 days earlier: __________________________________________________________

3 days earlier: __________________________________________________________

| **1a. MANUAL TASKS …**  **HEAVY LOADS** [4] | **Day** | **If yes, precisely describe manual task (type of task, load, duration and time). e.g. Lifted 50 large boxes, one at a time, (~15kg each box – perceived as heavy) from the floor and placed them on a bench at waist height. 8:00am - 20min.** |
| --- | --- | --- |
| **The first group of questions is about manual tasks.** Manual tasks include lifting, lowering, pushing, carrying or otherwise moving, holding or restraining any person, animal or item.  Firstly we are interested in manual tasks involving **HEAVY LOADS.**  So on the day of your back pain did you engage in any manual tasks involving a heavy load?  That was the (restate their description of the day).  Now what about the day before…. That was the (restate their description of the day).  OK now two days back That was the (restate their description of the day).  OK finally three days back That was the (restate their description of the day) | **Day of back pain** |  |
|  | □ Yes □ No |  |
|  | **Day before** |  |
|  | □ Yes □ No |  |
|  | **2 days earlier** |  |
|  | □ Yes □ No |  |
|  | **3 days earlier** |  |
|  | □ Yes □ No |  |

| **1b. MANUAL TASKS…**  **AWKWARD POSTURE** [4] | **Day** | **If yes, precisely describe manual task and posture (type of task, load, duration, time & body position). e.g. knelt down while gardening. 2:00pm – 40min.** |
| --- | --- | --- |
| Now I want you to think about manual tasks involving an **AWKWARD POSTURE.**  So on the day of your back pain did you engage in any manual tasks involving an **awkward position**?  That was the (restate their description of the day).  Now what about the day before…. That was the (restate their description of the day).  OK now two days back That was the (restate their description of the day).  OK finally three days back That was the (restate their description of the day) | **Day of back pain** |  |
|  | □ Yes □ No |  |
|  | **Day before** |  |
|  | □ Yes □ No |  |
|  | **2 days earlier** |  |
|  | □ Yes □ No |  |
|  | **3 days earlier** |  |
|  | □ Yes □ No |  |

| **1c. MANUAL TASKS…**  **AN OBJECT THAT COULD**  **NOT BE POSITIONED CLOSE**  **TO THE BODY** [4] | **Day** | **If yes, precisely describe manual task and posture (type of task, load, duration, time and body position). e.g. Lifted large box (~7Kg – perceived as light) out of the car boot and placed it on the floor. 11:00am – 10 sec.** |
| --- | --- | --- |
| Now I want you to think about manual tasks involving **AN OBJECT THAT COULD NOT BE POSITIONED CLOSE TO THE BODY.**  So on the day of your back pain did you engage in any manual tasks involving **an object that could not be positioned close to the body**? That was the (restate their description of the day).  Now what about the day before…. That was the (restate their description of the day).  OK now two days back That was the (restate their description of the day).  OK finally three days back That was the (restate their description of the day) | **Day of back pain** |  |
|  | □ Yes □ No |  |
|  | **Day before** |  |
|  | □ Yes □ No |  |
|  | **2 days earlier** |  |
|  | □ Yes □ No |  |
|  | **3 days earlier** |  |
|  | □ Yes □ No |  |

| **1d. MANUAL TASKS…**  **LIVE PEOPLE OR ANIMALS** [4] | **Day** | **If yes, precisely describe manual task and posture (type of task, load, duration, time and body position). e.g. Lifted 2 year-old child (~12kg – perceived as moderate) from the floor onto the bed. 8:00pm once. Lifted and carried baby (5 kg perceived as light), 3 to 4 times from one room to another. 8:10pm – 30min** |
| --- | --- | --- |
| Now I want you to think about manual tasks involving **LIVE PEOPLE OR ANIMALS.**  So on the day of your back pain did you engage in any manual tasks involving **live people or animals**? That was the (restate their description of the day).  Now what about the day before…. That was the (restate their description of the day).  OK now two days back…  That was the (restate their description of the day).  OK finally three days back…  That was the (restate their description of the day) | **Day of back pain** |  |
|  | □ Yes □ No |  |
|  | **Day before** |  |
|  | □ Yes □ No |  |
|  | **2 days earlier** |  |
|  | □ Yes □ No |  |
|  | **3 days earlier** |  |
|  | □ Yes □ No |  |

| **1e. MANUAL TASKS…**  **A LOAD THAT WAS**  **UNSTABLE, UNBALANCED OR**  **DIFFICULT TO GRASP OR**  **HOLD** [4] | **Day** | **If yes, precisely describe manual task and posture (type of task, load, duration, time and body position). e.g. lifted a 4 meter extension ladder (~12 Kg – perceived as moderate) from the car roof racks, carried to garage and hung on wall. 3:00pm - 5min.** |
| --- | --- | --- |
| Now I want you to think about manual tasks involving **A LOAD THAT WAS UNSTABLE, UNBALANCED OR DIFFICULT TO GRASP OR HOLD.**  So on the day of your back pain did you engage in any manual tasks involving **a load that was unstable, unbalanced or difficult to grasp or hold?**  That was the (restate their description of the day).  Now what about the day before…. That was the (restate their description of the day).  OK now two days back…  That was the (restate their description of the day).  OK finally three days back…  That was the (restate their description of the day) | **Day of back pain** |  |
|  | □ Yes □ No |  |
|  | **Day before** |  |
|  | □ Yes □ No |  |
|  | **2 days earlier** |  |
|  | □ Yes □ No |  |
|  | **3 days earlier** |  |
|  | □ Yes □ No |  |

| **2a. VIGOROUS**  **PHYSICAL ACTIVITY** | **Day** | **If yes, precisely describe activity/task, time and duration. e.g. ran 10km at fast pace (5 mins per km) 10:00am - 50min.** |
| --- | --- | --- |
| The next questions are about **VIGOROUS PHYSICAL ACTIVITY.** This could be sports or hobbies, paid or volunteer work, work outside the home and housework.  **Examples of vigorous** physical activity include: running, rope skipping, axe chopping, using heavy tools, canoeing and truck driving.  So on the day of your back pain did you engage in any manual tasks involving **VIGOROUS PHYSICAL ACTIVITIES?**  That was the (restate their description of the day).  Now what about the day before…  That was the (restate their description of the day).  OK now two days back… That was the (restate their description of the day).  OK finally three days back… That was the (restate their description of the day) | **Day of back pain** |  |
|  | □ Yes □ No |  |
|  | **Day before** |  |
|  | □ Yes □ No |  |
|  | **2 days earlier** |  |
|  | □ Yes □ No |  |
|  | **3 days earlier** |  |
|  | □ Yes □ No |  |

| **2b. MODERATE**  **PHYSICAL ACTIVITY** | **Day** | **If yes, precisely describe activity/task, time and duration. e.g. Mowed the lawn. 11:00am -12:00 pm.** |
| --- | --- | --- |
| The next questions are about **MODERATE PHYSICAL ACTIVITY.** This could be sports or hobbies, paid or volunteer work, work outside the home and housework.  **Examples of moderate** physical activity include: leisure cycling, fishing, general home repairs, music playing, golf, surfing and painting.  So on the day of your back pain did you engage in any manual tasks involving **MODERATE PHYSICAL ACTIVITIES?**  That was the (restate their description of the day).  Now what about the day before…. That was the (restate their description of the day).  OK now two days back… That was the (restate their description of the day).  OK finally three days back… That was the (restate their description of the day). | **Day of back pain** |  |
|  | □ Yes □ No |  |
|  | **Day before** |  |
|  | □ Yes □ No |  |
|  | **2 days earlier** |  |
|  | □ Yes □ No |  |
|  | **3 days earlier** |  |
|  | □ Yes □ No |  |

| **3. SLIP, TRIP OR FALL** [5] | **Day** | **If yes, precisely describe incident and time. e.g. Descending stairs, missed bottom step and jarred back. 8:30am – one occasion.** |
| --- | --- | --- |
| Now I want you to think about **SLIP, TRIP OR FALL.**  So on the day of your back pain did you have a **slip, trip or fall?** That was the (restate their description of the day).  Now what about the day before…. That was the (restate their description of the day).  OK now two days back…  That was the (restate their description of the day).  OK finally three days back….  That was the (restate their description of the day) | **Day of back pain** |  |
|  | □ Yes □ No |  |
|  | **Day before** |  |
|  | □ Yes □ No |  |
|  | **2 days earlier** |  |
|  | □ Yes □ No |  |
|  | **3 days earlier** |  |
|  | □ Yes □ No |  |

| **4. CONSUMED ALCOHOL** [6] | **Day** | **If yes, specify amount (refer to standard drink table at the end of booklet), time and duration. E.g. 2x red wine glasses (180ml). 8:20pm – 1h.** |
| --- | --- | --- |
| Now I want you to think about **ALCOHOL CONSUMPTION.**  So on the day of your back pain **did you consume alcohol?** That was the (restate their description of the day).  Now what about the day before…. That was the (restate their description of the day).  OK now two days back… That was the (restate their description of the day).  OK finally three days back… That was the (restate their description of the day) | **Day of back pain** |  |
|  | □ Yes □ No |  |
|  | **Day before** |  |
|  | □ Yes □ No |  |
|  | **2 days earlier** |  |
|  | □ Yes □ No |  |
|  | **3 days earlier** |  |
|  | □ Yes □ No |  |
|  | | |
| **5. SEXUAL ACTIVITY** [7] | **Day** | **If yes, specify time. E.g. 11:00pm** |
| Now I want you to think about **SEXUAL ACTIVITY.** So on the day of your back pain **did you engage in sexual activity?** That was the (restate their description of the day).  Now what about the day before…. That was the (restate their description of the day).  OK now two days back… That was the (restate their description of the day).  OK finally three days back… That was the (restate their description of the day) | **Day of back pain** |  |
|  | □ Yes □ No |  |
|  | **Day before** |  |
|  | □ Yes □ No |  |
|  | **2 days earlier** |  |
|  | □ Yes □ No |  |
|  | **3 days earlier** |  |
|  | □ Yes □ No |  |

| **6. DISTRACTION** [8] | **Day** | **If yes, specify time and duration, distraction and task.**  **e.g. Distracted by child crying while lifting a box from car boot and it slipped from his/her hands. 8:30am – one occasion.** |
| --- | --- | --- |
| Now I want you to think about being **DISTRACTED.**  So on the day of your back pain were you **DISTRACTED** for any reason while engaged in a task or activity? That was the (restate their description of the day).  Now what about the day before…. That was the (restate their description of the day).  OK now two days back… That was the (restate their description of the day).  OK finally three days back… That was the (restate their description of the day) | **Day of back pain** |  |
|  | □ Yes □ No |  |
|  | **Day before** |  |
|  | □ Yes □ No |  |
|  | **2 days earlier** |  |
|  | □ Yes □ No |  |
|  | **3 days earlier** |  |
|  | □ Yes □ No |  |
|  | | |
| **7. FATIGUE OR TIREDNESS** [9] | **Day** | **If yes, specify time and duration. e.g. Disrupted and poor sleep the night before as youngest child kept waking due to earache. 3:00am – 24h.** |
| Now I want you to think about **feeling FATIGUED or TIRED.** So on the day of your back pain did you feel **FATIGUED OR TIRED?** That was the (restate their description of the day).  Now what about the day before…. That was the (restate their description of the day).  OK now two days back… That was the (restate their description of the day).  OK finally three days back… That was the (restate their description of the day) | **Day of back pain** |  |
|  | □ Yes □ No |  |
|  | **Day before** |  |
|  | □ Yes □ No |  |
|  | **2 days earlier** |  |
|  | □ Yes □ No |  |
|  | **3 days earlier** |  |
|  | □ Yes □ No |  |

| **8. What do you think may have triggered your low back pain?**  **(Record what the patient thinks may have triggered his/her episode of back pain. eg. Bent down once to pick up newspaper from lawn. 7:00am - immediately).**  ___________________________________________________________________________________________  **____________________________________________________________________________________________**  **____________________________________________________________________________________________**  **____________________________________________________________________________________________**  **____________________________________________________________________________________________** | | |
| --- | --- | --- |
| So on the day of your back pain **did you do the activity described above?** That was the (restate their description of the day).  Now what about the day before…. That was the (restate their description of the day).  OK now two days back… That was the (restate their description of the day).  OK finally three days back… That was the (restate their description of the day) | **Day of back pain** |  |
|  | □ Yes □ No |  |
|  | **Day before** |  |
|  | □ Yes □ No |  |
|  | **2 days earlier** |  |
|  | □ Yes □ No |  |
|  | **3 days earlier** |  |
|  | □ Yes □ No |  |

**Reference**

1. Ware J, Sherbourne C: **The MOS 36-item short-form health survey (SF-36). 1. Conceptual framework and item selection**. *Medical Care* 1992, **30**:473-483.

2. Linton S, Hellsing A, Bergstrom G: **Exercise for workers with musculoskeletal pain: does enhancing compliance decrease pain?** *J Occup Med* 1996, **6**(3):177-190.

3. Armstrong T, Bauman A, Davies J: **Physical activity patterns of Australian Adults**. In*.* Edited by Welfare AIoHa. Canberra; 2000.

4. Australian Safety Compensation Council: **National code of practice for the prevention of musculoskeletal disorders from performing manual tasks at work**. In*.* Canberra; 2007.

5. Verma S, Lombardi D, Chang W, Courtney T, Huang Y, Brennan M, Mittleman M, Ware J, Perry M: **Rushing, distraction, walking on contaminated floors and risk of slipping in limited-service restaurants: a case--crossover study**. *Occupational & Environmental Medicine* 2010, **68**(8):575-581.

6. Vinson DC, Mabe N, Leonard LL, Alexander J, Becker J, Boyer J, Moll J: **Alcohol and injury. A case-crossover study**. *Arch Fam Med* 1995, **4**(6):505-511.

7. Dahabreh IJ, Paulus JK, Dahabreh IJ, Paulus JK: **Association of episodic physical and sexual activity with triggering of acute cardiac events: systematic review and meta-analysis**. *JAMA*, **305**(12):1225-1233.

8. Sorock G, Lombardi D, Peng D, Hauser R, Eisen E, Herrick R, Mittleman M: **Glove use and the relative risk of acute hand injury: a case-crossover study**. *Journal of Occupational & Environmental Hygiene* 2004, **1**(3):182-190.

9. Chen S, Fong P, Lin S, Chang C, Chan C: **A case-crossover study on transient risk factors of work-related eye injuries**. *Occupational & Environmental Medicine* 2009, **66**(8):517-522.
